# Sympathomimetic Activity of a *Hoodia gordonii* Product: A Possible Mechanism of Cardiovascular Side Effects

**DOI:** 10.1155/2013/171059

**Published:** 2013-11-06

**Authors:** Orsolya Roza, Norbert Lovász, István Zupkó, Judit Hohmann, Dezső Csupor

**Affiliations:** ^1^Department of Pharmacognosy, University of Szeged, Eötvös u. 6, Szeged 6720, Hungary; ^2^Department of Pharmacodynamics and Biopharmacy, University of Szeged, Eötvös u. 6, Szeged 6720, Hungary

## Abstract

*Hoodia gordonii*, a popular appetite suppressant, is widely used as an ingredient in many food supplements despite the fact that supporting scientific evidence is scarce. Recently alarming side effects of *H. gordonii* products (increased blood pressure and elevated pulse rate) have been reported. The aim of our study was to elucidate the underlying mechanism of these symptoms. A *H. gordonii*-containing product was tested for sympathomimetic activity. Isolated organ experiments on rat uterine rings revealed smooth muscle relaxant effect with a substantial component mediated through **β**-adrenergic receptors. Chromatographic comparison of the analyzed product and authentic plant material confirmed that the herbal product contained *Hoodia* spp. extract, and its cardiovascular effects may be linked to the compounds of the plant.

## 1. Introduction

According to WHO, 65% of the world's population live in countries where overweight and obesity kills more people than underweight. Obesity has become an epidemic, while safe and effective pharmacotherapy is still absent [1]. Numerous medications have been introduced for the treatment of obesity, which primarily act on the adrenergic, dopaminergic, cannabinoid, and serotonergic systems [2]. Because of serious side effects, there is only one drug (orlistat) in the European Union, and two in the United States (orlistat and topiramate + phentermine) approved for long-term use in the treatment of obesity; consequently, there is a disparity between demand and supply [3]. As a result, a broad scale of natural weight-loss products are under exploration, and countless products are currently available in the market with insufficient supporting scientific evidence [4].

*Hoodia gordonii* (Masson) Sweet ex Decne. (Apocynaceae) is a succulent plant consumed by Bushmen in South Africa in order to reduce appetite [5]. Its proposed active compound for weight-loss P57, an oxypregnane glycoside, was shown to cause increased ATP production in the hypothalamus after intracerebroventricular administration [6]. However, this mechanism of action is debatable, as there was no detectable P57 in the brain after oral administration in CD1 female mice according to Madgula et al. [7]. Another possible explanation for its appetite suppressant properties is that P57 may also act on the bitter taste receptors resulting in the secretion of CCK [8].

Besides P57, numerous other pregnane glycosides, based on five aglycones, were identified as main constituents, of which 19 are glycosides of hoodigogenin A, a unique pregnane derivate that can be found only in *Hoodia* species [9–11]. Thirteen calogenin glycosides were also reported, as well as one compound with isoramanone aglycone [11, 12]. Furthermore, two constituents were isolated as the first two naturally occurring glycosides of 5(6→7) *abeo*-sterol aglycones, namely, hoodistanal and dehydrohoodistanal [11]. Further components belonging to the compound classes of sterols, fatty acids, and alcohols were detected in low concentrations using GC-FID and GC-MS methods [13]. *Hoodia* species are stapeliads, emitting fetid scents; thus, *H. gordonii* flower volatiles were studied, and 94 compounds were also reported [14].

Although controversial appetite-suppressant effects have been demonstrated in some *in vivo* experiments conducted on rats and chickens, human evidence is still lacking [6, 15–17]. The only available human clinical study reported no change in body weight or energy expenditure; however, administration of a purified *H. gordonii* extract was associated with significant increase in blood pressure and pulse rate [18]. Similar side effects have been reported to the Department of Pharmacognosy, University of Szeged, by consumers of a product available in the Hungarian market (Hoodia spray). Taking into consideration these alarming side effects and the popularity of* H. gordonii* among natural products used for weight management, the unknown mechanisms behind these side effects seemed to be worthy of detailed investigation.

Numerous effective weight loss drugs, like sibutramine or amphetamine derivatives were withdrawn from the market because of their serious side effects, including increased blood pressure and elevated pulse rate. Concerning that the chemical composition of *Hoodia* spp. is not yet fully mapped, it is also possible that a group of compounds other than pregnane glycosides may be responsible for the reported side effects or for the appetite suppressant activity of *H. gordonii* [9–12].

Our present work has focused on the effect of the above-mentioned Hoodia spray on *β*-adrenergic receptors in rat uterus to explore the potential role of *β*-adrenergic receptor agonist activity in the possible cardiovascular adverse effects of the plant. Adulteration among diet and weight management supplements occurs frequently by using active pharmaceuticals as adulterants to intensify the anticipated effect [19, 20]. Synthetic weight loss drugs also possess cardiovascular side effects; thus, we performed analytical comparison with authentic plant material and also tested the product for the presence of usually occurring adulterants. Mass spectrometry was applied to confirm the presence of P57, a marker compound of *Hoodia* species, and HPLC fingerprinting was also performed for comparison to confirm the authenticity of the product.

## 2. Materials and Methods

### 2.1. Materials

The aerial parts of* Hoodia gordonii* (Masson) Sweet ex Decne. were obtained from a local succulent plant nursery in Szeged, Hungary, and authenticated by one of the authors (Orsolya Roza). A voucher specimen (no. 812) has been preserved in the Herbarium of the Department of Pharmacognosy, University of Szeged, Szeged, Hungary. Fytofontana Dietceutical Hoodia spray (distributed by Herb-Pharma Hu Ltd., Budapest, Hungary), a medical device registered in Hungary, was bought in a local pharmacy in Szeged, Hungary. The composition of the product displayed on the package is the following: *Hoodia gordonii* extract (corresponding to 2.2 g plant material/2 mL solution), peppermint, and alcohol. Amphetamine and methamphetamine were obtained from Scientific Section, DSRRB/DOA/UNDCP (Vienna, Austria). Sibutramine was extracted previously in our lab [21]. Ephedrine was purchased from Phoenix Pharma Zrt (Budapest, Hungary).

### 2.2. Preparation of Extracts

Authentic *Hoodia* extract for HPLC and HPTLC comparison was prepared from 7 g fresh, ground plant material with 3 × 25 mL of AcNi, using ultrasonic bath (15 min). After filtration, the solution was evaporated to dryness under reduced pressure to yield 0.15 g of extract. The residue was dissolved in 2 mL of MeOH, filtered, and then evaluated by HPLC and HPTLC.

The *Hoodia* product contained alcohol, and since ethanol may alter *β*-adrenergic receptor binding affinity and smooth muscle contractility [22], 25 mL spray was evaporated to dryness, then the residue was dissolved to the original volume with physiological saline-dimethyl sulfoxide (95 : 5) and then filtered to perform organ baths studies.

### 2.3. High Pressure Liquid Chromatography

Chromatographic analyses of the authentic *H. gordonii* extract and the commercial product were performed on a Waters HPLC system (Waters Associates, Milford, MA, USA) with a Waters 600 controller and pump, equipped with a 2487 dual absorbance detector. The separation was carried out on a Kinetex XB-C18 (2.6 *μ*m, 100 Å, 100 × 4.6 mm) column (Phenomenex, Torrance, USA), operated at 40°C. Injection volume of 20 *μ*L was used. Chromatographic elution was accomplished by gradient solvent system consisting of MeOH, AcNi, and H_2_O, according to the method published by Janssen et al. [23].

### 2.4. Thin Layer Chromatography

Thin layer chromatographic analyses were carried out on aluminium TLC sheets (20 × 20 cm), coated with silica gel 60 F_254_ (Merck KgaA, Darmstadt, Germany). For each test, 10 *μ*L of Hoodia spray and standard solutions were applied. For sibutramine a solvent system of toluene-MeOH 95 : 5 was used for development. After drying, visualization was obtained by spraying with Dragendorff reagent. For methamphetamine and amphetamine a mobile phase of MeOH-cc. NH_3_ 100 : 1.5 was used. After spraying with Marquis reagent (cc. H_2_SO_4_-formalin 20 : 1.5) and heating at 110°C for 2 min, detection was performed under UV light (366 nm) [24]. Ephedrine identification was carried out according to the Ph. Eur. 5, using a solvent system of CH_2_Cl_2_-cc. NH_3_-2-propanol 5 : 15 : 80 and ninhydrin reagent for spraying. The plate was heated at 110°C for 5 min for visualization.

### 2.5. HPTLC

HPTLC comparison of the product and the authentic *Hoodia* extract was carried out according to the method described by Rumalla et al., using silica gel coated 60 F_254_ HPTLC plates (10 × 20 cm) (Merck KgaA, Darmstadt, Germany) [25]. CHCl_3_-MeOH-H_2_O 70 : 30 : 3 as mobile phase and anisaldehyde as visualization reagent were used. After drying, plates were heated at 110°C for 5 min.

### 2.6. Mass Spectrometry

Analyses were performed on an API 2000 triple quadrupole tandem mass spectrometer (AB Sciex Instruments, Foster, CA, USA). Data integration was performed with Analyst 1.5.2 software version (AB Sciex Instruments). Electrospray ionization interface was used with the source temperature of 250°C, operating in positive mode using multiple reaction monitoring (MRM). The following conditions were used: capillary voltage 5500; curtain gas pressure 10 psi; collusion gas pressure was 6 psi; nebulizer gas pressure 50 psi; auxiliary gas pressure 20 psi.

### 2.7. Animals

Animals were treated in accordance with the Directive of the Council of the European Communities (86/609/ECC) and the Hungarian Act XXVIII of 1998 on the Protection of Animals in Research (Section 32). Experiments involving animal subjects were carried out with the approval of the Hungarian Ethical Committee for Animal Research (registration number: IV./01758-2/2008). Sexually mature female Sprague-Dawley rats (body mass: 140–160 g, 50–60 days old) were mated in the early morning hours. Copulation was confirmed by the presence of a copulation plug or spermatozoa in the vagina. The day of copulation was considered to be the first day of pregnancy. The animals were housed in temperature- (20–23°C), humidity- (40–60%), and light- (12 h of light, 12 h of dark) regulated rooms, with water and standard rodent food (Bioplan Ltd., Isaszeg, Hungary) intake available *ad libitum*.

### 2.8. *In Vitro* Organ Bath Studies

Uteri were removed from nonpregnant rats in the estrus phase and from pregnant rats on day 22 of gestation. Five mm long muscle rings were sliced from the uterine horns and mounted vertically in an organ bath containing 10 mL of de Jongh solution (composition: 137 mM NaCl, 3 mM KCl, 1 mM CaCl_2_, 1 mM MgCl_2_, 12 mM NaHCO_3_, 4 mM NaH_2_PO_4_, and 6 mM glucose, pH = 7.4). The organ bath was maintained at 37°C, and carbogen (95% O_2_ + 5% CO_2_) was bubbled through it. After mounting, the rings were equilibrated for 1 h before the experiments were undertaken, and the de Jongh solution was changed every 15 min. The initial tension of the preparation was set to about 1.5 g, which was relaxed to about 0.5 g at the end of equilibration. The tension of the myometrial rings was measured with a gauge transducer (SG-02; Experimetria Ltd., Budapest, Hungary) and recorded with SPEL Advanced ISOSYS Data Acquisition System (Experimetria Ltd., Budapest, Hungary) [26]. The uterus-relaxant effect of Hoodia spray was investigated on spontaneous and 25 mM KCl-induced contractions alone and in the presence of 10 *μ*M propranolol. The extract was administered cumulatively (50 *μ*L, 50 *μ*L, 100 *μ*L, 200 *μ*L, and 400 *μ*L) into the organ bath, and the effect of the solvent system was excluded in separate experiments. The tissue samples were incubated for 5 min with each concentration. Areas under the curves (AUCs) of 5-min periods were evaluated; the effect of the Hoodia preparation was expressed as percentages of the spontaneous and KCl-induced contractions preceding the administration of the tested substance. All experiments were carried out on at least 6 animals, and each reported value is given as the mean ± SEM. Unpaired *t*-test was used for statistical evaluation of the presence of propranolol. All calculations and statistical analyses were performed with Prism 5.0 computer software (Graph Pad Software Inc., San Diego, CA, USA).

## 3. Results and Discussion

### 3.1. HPLC and HPTLC Comparison of the Product and Extract

**Figure 1:**
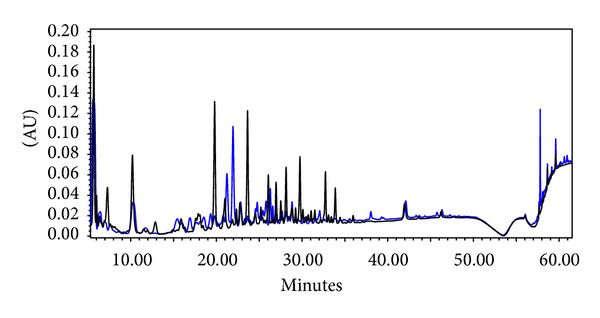
HPLC fingerprint chromatograms of *Hoodia* extract (blue) and Hoodia spray (black).

The HPLC chromatogram of the plant extract and the product showed similarity, having similar fingerprints between 16 and 36 min at 220 nm (Figure 1). Differences in peak heights and areas can be explained with the dissimilarity of extraction methods (in the product's leaflet there was no referral to the extraction method applied). As other *Hoodia* species contain the same constituents, but in different ratios, the chance that *Hoodia* species other than *H. gordonii* being used in the preparation cannot be excluded [25]. In the chromatogram of the product, few other peaks occurred, possibly due to additives present in the spray, such as *Mentha piperita*. Regarding the colors and retention factors of the detected spots, HPTLC analysis (not shown) also confirmed the similarity of the product and the plant extract.

### 3.2. TLC Analyses for Adulterants

Amphetamine, methamphetamine, sibutramine, and ephedrine were not detected in the product. 

### 3.3. Mass Spectrometric Authentication

The product was also tested for P57 by mass spectrometry in multiple reaction monitoring (MRM) mode. This method allows the reliable identification of molecules with known *m/z* values in complex mixtures, such as herbal extracts. In our experiments, P57, a characteristic compound of *Hoodia*, was chosen for mass spectrometric identification. LiCl_3_ was added to the sample, to enhance sensitivity. Both *m/z* 311.3 and *m/z* 785.5 product ions were detected with the precursor ion *m/z *885.5, indicating that P57 is present in the Hoodia spray. The aglycone was identified too, with the product ions of *m/z *319.3 and *m/z *337.3. All of these transitions have been reported in the literature for P57 [27].

### 3.4. *In Vitro* Organ Bath Studies

**Figure 2:**
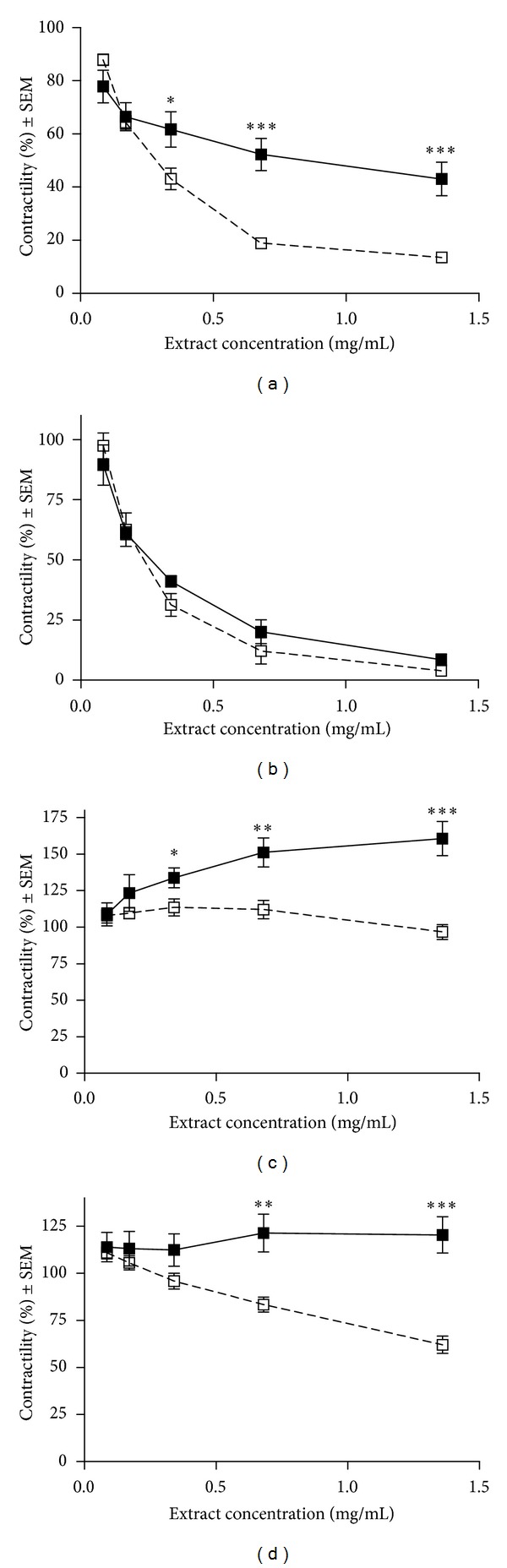
Effects of Hoodia spray on spontaneous ((a) and (c)) and KCl-stimulated ((b) and (d)) contractility of uterine rings from nonpregnant ((a) and (b)) and late-pregnant ((c) and (d)) animals. Experiments were performed in the presence (■) and absence (□) of propranolol. *, **, and *** indicate *P* < 0.05, *P* < 0.01, and *P* < 0.001, respectively.

*β*-adrenergic agonists are reported to decrease food intake and exhibit anorectic properties [28]. Stimulation of *β*-adrenoceptors also results in different cardiovascular symptoms. Since cardiovascular side effects have been reported for *H. gordonii* extracts, the stimulation of *β*-adrenergic receptors seems to be a rational explanation for the mechanism of its anorectic action [18]. Both *α*- and *β*-adrenergic receptors play crucial roles in the motor activity of myometrial smooth muscle eliciting contraction and relaxation, respectively, rendering uterine preparations ideal for investigations of drugs with actions on the sympathetic system. In the presented set of *in vitro* organ bath experiments, the myometrial effects of the *H. gordonii* extract-containing product were monitored against spontaneous and KCl-stimulated contractions of uterine rings from nonpregnant and late-pregnant (day 22) rats. In order to separate the actions mediated through *α*- and *β*-adrenoceptors all experiments were performed with and without nonselective *β*-antagonist propranolol (10 *μ*M). The product elicited a marked and concentration-dependent relaxation against both spontaneous and stimulated contractions. The inhibition of spontaneous contractility was significantly decreased in the presence of propranolol (Figure 2). The relaxation of KCl-stimulated uteri was not modified in the presence of propranolol. The relaxing effect of the spray was substantially less pronounced on uteri from late-pregnant animals, but propranolol significantly modified it. The gestation-dependent alteration in the myometrial action of substances acting on the sympathetic system could be explained by the changes in the receptor function during pregnancy. The nonpregnant uterus of the rat exhibits limited *α*-adrenoceptor-mediated contraction but efficiently relaxed by *β*-adrenergic stimulation. Late-pregnant myometrium is sensitive to both *α*- and *β*-adrenergic stimulation, and therefore, the overall response is determined by the receptor preference of the tested substance [26]. It is plausible that the limited relaxation of late-pregnant myometrium induced by the product is a consequence of a balanced *α*- and *β*-adrenoceptor stimulation which can be shifted towards increased contractility by blocking the action mediated through *β*-receptors. 

Based on the propranolol-sensitive component of the uterine action of the product, a sympathomimetic effect with substantial *β*-receptor-mediated contribution is proposed. Our results support the cardiovascular side effects reported in a human clinical trial. 

As we have shown, Hoodia spray possesses sympathomimetic effects, which can manifest in both appetite suppression and increased thermogenesis, resulting in weight loss [29, 30]. Moreover, in the experiments performed in the US patent application, increased water consumption was recorded during feeding studies, which is a well-known effect of sympathomimetic agents, let alone the side effects, elucidated in the human clinical trial [17]. Chemical composition of *Hoodia* is not yet fully mapped. In the literature, primarily pregnane glycosides are in the focus; however, other potentially active constituents with sympathomimetic activity may also be present in the plant.

Sympathomimetic agents as amphetamine derivatives or sibutramine are effective in weight management, but due to their serious cardiovascular side effects, they were withdrawn from the market. In the case of *H. gordonii*, cardiovascular side effects and proposed anorectic property are reported, raising the questions whether these effects are caused by the same constituents; and if they are, whether the responsible compound is truly P57.

## 4. Conclusion

We have shown for the first time that a *H. gordonii*-containing product has sympathomimetic activity on rat uterus mediated through *β*-adrenergic receptors. HPLC and HPTLC tests were performed to compare the product with authentic plant material. Due to the frequent adulteration of food supplements, we also tested the Hoodia spray product for the presence of some frequently used adulterants having sympathomimetic property. Our experiments revealed that the analyzed product did contain *Hoodia* species extract and was void of known adulterants with sympathomimetic activity (amphetamine, methamphetamine, sibutramine, and ephedrine). Considering the large number of *Hoodia*-containing products in the market, the presented data should be regarded alarming, due to possible cardiovascular side effects of these supplements.

## References

[1] http://www.who.int/mediacentre/factsheets/fs311/en/index.html.

[2] Kennett GA, Clifton PG (2010). New approaches to the pharmacological treatment of obesity: can they break through the efficacy barrier?. *Pharmacology Biochemistry and Behavior*.

[3] Kangand JG, Park CY (2012). Anti-obesity drugs: a review about their effects and safety. *Diabetes & Metabolism Journal*.

[4] Yun JW (2010). Possible anti-obesity therapeutics from nature—a review. *Phytochemistry*.

[5] van Heerden FR (2008). Hoodia gordonii: a natural appetite suppressant. *Journal of Ethnopharmacology*.

[6] MacLean DB, Luo L-G (2004). Increased ATP content/production in the hypothalamus may be a signal for energy-sensing of satiety: studies of the anorectic mechanism of a plant steroidal glycoside. *Brain Research*.

[7] Madgula VLM, Ashfaq MK, Wang Y-H (2010). Bioavailability, pharmacokinetics, and tissue distribution of the oxypregnane steroidal glycoside P57AS3 (P57) from Hoodia gordonii in mouse model. *Planta Medica*.

[8] Nevé BL, Foltz M, Daniel H, Gouka R (2010). The steroid glycoside H.g.-12 from Hoodia gordonii activates the human bitter receptor TAS2R14 and induces CCK release from HuTu-80 cells. *American Journal of Physiology—Gastrointestinal and Liver Physiology*.

[9] Pawar RS, Shukla YJ, Khan SI, Avula B, Khan IA (2007). New oxypregnane glycosides from appetite suppressant herbal supplement Hoodia gordonii. *Steroids*.

[10] Dall’Acqua S, Innocenti G (2007). Steroidal glycosides from Hoodia gordonii. *Steroids*.

[11] Shukla YJ, Pawar RS, Ding Y, Li X-C, Ferreira D, Khan IA (2009). Pregnane glycosides from Hoodia gordonii. *Phytochemistry*.

[12] Pawar RS, Shukla YJ, Khan IA (2007). New calogenin glycosides from Hoodia gordonii. *Steroids*.

[13] Russell PJ, Swindells C (2012). Chemical characterisation of Hoodia gordonii extract. *Food and Chemical Toxicology*.

[14] Jürgens A, Dötterl S, Meve U (2006). The chemical nature of fetid floral odours in stapeliads (Apocynaceae-Asclepiadoideae-Ceropegieae). *New Phytologist*.

[15] van Heerden FR, Marthinus Horak R, Maharaj VJ, Vleggaar R, Senabe JV, Gunning PJ (2007). An appetite suppressant from Hoodia species. *Phytochemistry*.

[16] Mohlapo TD, Ng’ambi JW, Norris D, Malatje MM (2009). Effect of Hoodia gordonii meal supplementation at finisher stage on productivity and carcass characteristics of Ross 308 broiler chickens. *Tropical Animal Health and Production*.

[17] van Heerden FR, Vleggaar R, Horak RM, Learmonth RA, Maharaj VJ, Whittal RD Pharmaceutical compositions having appetite-suppressant activity.

[18] Blom WAM, Abrahamse SL, Bradford R (2011). Effects of 15-d repeated consumption of Hoodia gordonii purified extract on safety, ad libitum energy intake, and body weight in healthy, overweight women: a randomized controlled trial. *American Journal of Clinical Nutrition*.

[19] Champagne AB, Emmel KV (2011). Rapid screening test for adulteration in raw materials of dietary supplements. *Vibrational Spectroscopy*.

[20] Jung J, Hermanns-Clausen M, Weinmann W (2006). Anorectic sibutramine detected in a Chinese herbal drug for weight loss. *Forensic Science International*.

[21] Csupor D, Boros K, Danko B, Veres K, Szendrei K, Hohmann J (2013). Rapid identification of sibutramine in dietary supplements using a stepwise approach. *Die Pharmazie*.

[22] Valverius P, Hoffman PL, Tabakoff B (1987). Effect of ethanol on mouse cerebral cortical *β*-adrenergic receptors. *Molecular Pharmacology*.

[23] Janssen H-G, Swindells C, Gunning P (2008). Quantification of appetite suppressing steroid glycosides from Hoodia gordonii in dried plant material, purified extracts and food products using HPLC-UV and HPLC-MS methods. *Analytica Chimica Acta*.

[24] Division des Stupéfiants Méthodes recommandées pour l'identification de l'amphétamine et de la métamphétamine.

[25] Rumalla CS, Avula B, Shukla YJ (2008). Chemical fingerprint of Hoodia species, dietary supplements, and related genera by using HPTLC. *Journal of Separation Science*.

[26] Csik G, Spiegl G, Minorics R, Falkay G, Zupko I (2010). The effects of adjuvant arthritis on the myometrial adrenergic functions in the nonpregnant and the late-pregnant rat. *Journal of Physiology and Pharmacology*.

[27] van Platerink CJ, Janssen H-GM, Graf B, Abrahamse L, Haverkamp J (2011). Quantification of steroid glycosides from Hoodia gordonii in porcine plasma using high performance liquid chromatography-mass spectrometry. *Journal of Chromatography B*.

[28] Fernández-López J-A, Remesar X, Foz M, Alemany M (2002). Pharmacological approaches for the treatment of obesity. *Drugs*.

[29] Landsberg L, Saville ME, Young JB (1984). Sympathoadrenal system and regulation of thermogenesis. *The American Journal of Physiology*.

[30] Lowell BB, Spiegelman BM (2000). Towards a molecular understanding of adaptive thermogenesis. *Nature*.

